# Cytostatic and Cytotoxic Effects of Hollow-Shell Mesoporous Silica Nanoparticles Containing Magnetic Iron Oxide

**DOI:** 10.3390/nano11092455

**Published:** 2021-09-21

**Authors:** Manuel Pérez-Garnes, Victoria Morales, Raul Sanz, Rafael A. García-Muñoz

**Affiliations:** Department of Chemical and Environmental Technology, Rey Juan Carlos University, C/Tulipán s/n, 28933 Madrid, Spain; noningarnes@hotmail.com (M.P.-G.); victoria.morales@urjc.es (V.M.)

**Keywords:** hollow-shell mesoporous silica nanoparticles, iron oxide magnetic nanoparticles, drug delivery systems, toxicity, drug-structure-directing agent (DSDA)

## Abstract

Among the different types of nanoparticles used in biomedical applications, Fe nanoparticles and mesoporous siliceous materials have been extensively investigated because of their possible theranostic applications. Here, we present hollow-shell mesoporous silica nanoparticles that encapsulate iron oxide and that are prepared using a drug-structure-directing agent concept (DSDA), composed of the model drug tryptophan modified by carbon aliphatic hydrocarbon chains. The modified tryptophan can behave as an organic template that allows directing the hollow-shell mesoporous silica framework, as a result of its micellisation and subsequent assembly of the silica around it. The one-pot synthesis procedure facilitates the incorporation of hydrophobically stabilised iron oxide nanoparticles into the hollow internal silica cavities, with the model drug tryptophan in the shell pores, thus enabling the incorporation of different functionalities into the all-in-one nanoparticles named mesoporous silica nanoparticles containing magnetic iron oxide (Fe_3_O_4_@MSNs). Additionally, the drug loading capability and the release of tryptophan from the silica nanoparticles were examined, as well as the cytostaticity and cytotoxicity of the Fe_3_O_4_@MSNs in different colon cancer cell lines. The results indicate that Fe_3_O_4_@MSNs have great potential for drug loading and drug delivery into specific target cells, thereby overcoming the limitations associated with conventional drug formulations, which are unable to selectively reach the sites of interest.

## 1. Introduction

Over the past few decades, nanotechnology has emerged as a discipline with multiple applications in fields such as energy, environment, optics, and biomedicine [1,2,3,4,5,6,7,8]. Nanoparticles (NPs) have been investigated for biomedical applications because their versatility enables the development of smart materials that can target specific tissues and because of their potential as drug vehicles. Thus, the current trends of employing NPs in medicine are focused on the development of novel strategies for the treatment and diagnosis of several diseases [9]. Nowadays, NPs for biomedical applications are obtained via different strategies, including organic systems (micelles, liposomes, and polymers, among others) [10,11], inorganic-based NPs (silica, iron oxides, carbon nanotubes, etc.) [12,13,14], and organic–inorganic systems [15].

Iron oxide NPs have received the attention of several studies because of their feasibility of synthesis and their possibilities of obtaining magnetic NPs. Thus, magnetite (Fe_3_O_4_), maghemite (γ-Fe_2_O_3_), and hematite (α-Fe_2_O_3_) have been widely investigated for the diagnosis and treatment of different types of cancer [16]. Specifically, magnetite NPs show ferromagnetic behaviour for sizes greater than 20 nm, while below this threshold, they possess superparamagnetic properties, with magnetisation values between 25 and 180 emu/g [17]. These properties make magnetite NPs excellent nanodevices, such as contrast agents in magnetic resonance imaging (MRI), therapeutic agents to be applied in magnetic hyperthermia, and drug nanocarriers in drug delivery systems [18,19,20]. Usually, the superparamagnetic NPs are functionalised with molecules to avoid their agglomeration in a physiological medium, as well as to improve their biocompatibility and to enhance their bioaccumulation in a specific target tissue, allowing a controlled drug delivery [9,21,22,23]. Therefore, several researchers have investigated magnetite NPs combined with therapeutic agents, such as drugs for targeted transportation and selective release, siRNA for gene therapy, functional moieties to facilitate passage through cell membranes, photofunctional moieties, etc. [9,24]. In addition to these applications, superparamagnetic NPs can be employed in various treatments—mainly cancer hyperthermia and immunotherapy—by two main routes, leading to an increase in thermal energy: magnetic hyperthermia by the application of an alternating magnetic field and photothermal ablation by the irradiation and adsorption of light [25,26,27,28,29]. 

However, these iron oxide magnetic NPs present some problems related to their size and shape, among others, for use in biomedical applications. Thus, the cytotoxicity of iron oxide NPs with different sizes has been investigated, and the results showed that NPs below 6 nm had a negligible effect, while larger NPs induced some cytotoxicity [30]. Also, the size and shape of magnetic NPs are key factors for the time that they can circulate in blood vessels [31,32,33]. Additionally, bare iron oxide magnetic NPs tend to aggregate because of their affinity, permitting a rapid uptake by clearance organs, which results in a short lifetime. Surface coating or modification of iron oxide NPs is required to overcome these limitations. Thus, different strategies are used depending on the application and target tissue, are used. To prevent the aggregation of iron oxide magnetic NPs, different coating materials have been investigated, including oleic acid, cetyltrimethylammonium bromide (CTAB), citric acid, polymers, aminosilanes, silica, amino acids, and unspecific proteins [34,35,36,37,38,39]. One well-reported method for coating iron oxide magnetic NPs is the grafting of polymeric chains onto the NPs’ surface. Polymers can decrease the inherent negative charge on the surface of NPs, so they can strongly affect the blood circulation, cytotoxicity, cellular endocytosis, and biodegradation of NPs. In addition, the polymer coverage may provide a framework for the physical adsorption or chemical bonding of active molecules, such as drugs and RNA and DNA strands. For these purposes, a wide range of polymers have been studied, including polyethylene glycol [40], dextran [41], chitosan [42], polyacrylic acid [43], and carboxymethylcellulose [44]. On the other hand, coating NPs with silica, producing so-called mesoporous silica nanoparticles (MSNs), is a promising methodology. MSNs are proven to be mechanically and chemically stable nanocarriers, containing mesopores that are suitable drug delivery systems. Iron oxide magnetic NPs coated with MSNs are core–shell magnetic NPs with an encapsulated magnetic core and a porous shell. These NPs are easy to functionalise, are able to incorporate cargo drugs, and can be modulated and stimulated by magnetic interactions to selectively accumulate and release the drugs [45,46,47,48]. Different routes can be used for the silica coating of iron oxide magnetic NPs. Stöber’s method is one of the most employed methods for the coating of metallic NPs and consists of a sol-gel process from a silica source in an alcoholic medium using NH_4_OH as a catalyst [49,50,51]. Employing methods derived from Stöber’s method, several studies have been conducted to obtain silica materials with different morphologies containing magnetite cores. The silica shell can be tailored by varying the amount of the silica source and catalyst in the media [13,52,53].

In the present work, we propose an oil-in-water method for encapsulating iron oxide magnetic NPs within hollow-shell MSNs (h-MSNs), prepared using the drug-structure-directing agent (DSDA) concept [12,54]. By this method, an amino acid, L-tryptophan, is conveniently modified with fatty acids by means of amidation reactions, leading to an anionic DSDA. This DSDA, together with cationic aminosilane moieties, acts as a co-structure-directing agent (CSDA) to guide the formation of the h-MSN structure. Furthermore, iron oxide magnetic NPs (Fe_3_O_4_ NPs)—stabilised with different molecules—are added to the h-MSN synthesis media during the sol-gel process, leading to the formation of all-in-one hollow-shell MSNs with magnetic cores and loaded with the model drug tryptophan (Fe_3_O_4_@MSNs). Additionally, the cell viability and antiproliferative activity reveal the absence of cytotoxicity, which makes these nanocarriers potential candidates for selectively and safely delivering drugs into cancer cells.

## 2. Materials and Methods

### 2.1. Materials

L-tryptophan (98%), decanoyl chloride (98%), sodium hydroxide (97%), sodium bicarbonate (97%), 3-aminopropyl trimethoxysilane (97%), tetraethyl orthosilicate (98%), butyric acid (99%), cetyltrimethylammonium bromide (99%), and dichloromethane (99.8%) were purchased from Sigma-Aldrich (St. Louis, MO, USA). Tetrahydrofuran (TFH, 99.5%), hydrochloric acid (35% *w/w*), oleic acid (99%), palmitic acid (99%), citric acid (99%), ammonia solution (32% *w/w*), iron (III) chloride (97%), iron (II) chloride tetrahydrate (99%), and sodium chloride (99.5%) were supplied by Scharlab (Barcelona, Spain).

### 2.2. Synthesis and Functionalisation of Magnetic NPs (Fe_3_O_4_ NPs)

The iron oxide magnetite NPs (Fe_3_O_4_ NPs) were synthesised using a co-precipitation method, adding the ferric salts FeCl_2_ and FeCl_3_ in a molar ratio of 1:2 of Fe^+2^:Fe^+3^ in an aqueous medium using NH_4_OH as a base. Briefly, 2.4 g of FeCl_3_ and 6.0 g of FeCl_2_·4H_2_O were dissolved in water at 80 °C in a nitrogen atmosphere. After homogenisation, 20 mL of NH_4_OH (28–30% NH_3_ basis in water) was added dropwise, and the reaction was kept overnight. The NPs were collected by filtration, washed several times with water and ethanol, and dried and stored in vacuum.

Fe_3_O_4_ NPs were stabilised for their subsequent incorporation into the silica framework with different moieties: oleic acid, palmitic acid, butyric acid, citric acid, 3-aminopropyltrimethoxysilane (APS), and cetyltrimethylammonium bromide (CTAB). The molar ratio between Fe_3_O_4_ NPs and the different stabilisers was maintained at a constant ratio of 1:1.

### 2.3. Synthesis of Magnetic MSNs (Fe_3_O_4_@MSNs)

First, the DSDA surfactant, N-decanoyl-L-tryptophan (NDLT), was prepared as described previously [11]. Briefly, 4.5 g of L-tryptophan was dissolved in 50 mL of water and 25 mL of THF. The resulting solution was cooled at 0 °C in an ice bath, and 3 g of NaOH was added and stirred for 30 min until the L-tryptophan was completely dissolved. Then, 4.24 mL of decanoyl chloride was added dropwise over a period of 10 min and dissolved in 50 mL of the THF. The reaction was maintained in the ice bath for 2 h and then overnight at room temperature. The product was acidified using 12 mL of HCl (37% *w/w*), and the THF was removed by low pressure, and the product was extracted with dichloromethane. The dichloromethane was removed by low pressure, and the final product was dried. The Fe_3_O_4_@MSNs were prepared in an aqueous solution containing the corresponding DSDA and stabilised Fe_3_O_4_ NPs in a molar ratio of DSDA:Fe_3_O_4_ NPs:water of 1:0.6:2000. After the complete dispersion of both components, APS was added as a co-surfactant; after 5 min, tetraethyl orthosilicate (TEOS) was added as the main silica source. The final molar ratio of NDLT:Fe_3_O_4_ NPs:APS:TEOS:water was 1:0.6:1.65:11.8:2000. Briefly, the DSDAs were dispersed in water overnight at 80 °C, then the dispersion was cooled to 60 °C and the stabilised Fe_3_O_4_ NPs were added to the aqueous solutions. After 30 min of mechanical stirring, the APS was added and stirred for 5 min; after that, TEOS was added dropwise and stirred for an additional 10 min. The solution was kept without stirring for 24 h at 60 °C and for an additional 72 h at 100 °C. The materials were recovered by filtration and washed with abundant de-ionised water and dried under vacuum.

### 2.4. Physicochemical Characterisation

The organic content (DSDA and APS) in the Fe_3_O_4_@MSNs was determined by thermogravimetry from ambient temperature to 800 °C at 5 °C/min under an air atmosphere using a Star System Mettler Thermobalance. X-ray diffraction patterns were recorded on a Philips X’PERT MPD powder diffractometer equipped with CuKα radiation. The textural properties were obtained by N_2_ adsorption–desorption isotherms at −196 °C with a Micromeritics TriStar 3000 instrument (Norcross, GA, USA) from the calcined and extracted Fe_3_O_4_@MSN samples. Before measurement, the materials were outgassed at 300 °C with an N2 flux. Slit pore geometry was assumed for the calculation of the mesopores’ size distribution using the NLDFT model. Transmission electron microscopy (TEM) images of the samples were recorded using a Philips Tecnai F20 microscope (Amsterdam, Netherlands) operating at 200 kV. Previously, the samples were crushed, dispersed in acetone, and deposited on a carbon-coated copper grid. The measurements of the NPs’ features were derived from the TEM images using ImageJ software. Images of the surfaces of the Fe_3_O_4_@MSNs were collected by scanning electron microscopy (SEM) using a Nova NanoSEM 230 machine. STEM images and elemental mapping of the samples were recorded using a JEOL JEM200F (Tokyo, Japan) operated at 200 kV with a 0.19 nm resolution, equipped with a HAADF detector and 100 mm^2^ JEOL CENTURIO EDS detector (Tokio, Japan). A NanoPlus DLS Zeta Potential from Micromeritics was used for obtaining the hydrodynamic size of nanoparticles values of the particle suspensions. The CONTIN method has been used to resolve particle size distributions from the measured autocorrelation functions.

### 2.5. DSDA Delivery and Cell Viability Studies

The release of the surfactant, containing L-tryptophan, from Fe_3_O_4_@MSN materials was monitored at different times (up to 3 months) from a sample of 100 mg placed into dialysis bags (cut-off 10,000 Da) in 100 mL of the medium at pH 8 (0.1 M of NaHCO_3_ in water) at 37 °C under sealed conditions. The concentrations of the released surfactant were calculated using the Lambert–Beer law according to the absorbance of the surfactants by UV-spectrometry (JASCO V-630, Tokyo, Japan) at 280 nm, using the corresponding calibration curve.

Human colon (DLD-1 and HCT 116) cancer cells were obtained from the American Type Culture collection. Cells were cultured in DMEM, supplemented with 10% fetal bovine serum, and maintained under standard conditions of temperature (37 °C), humidity (95%), and carbon dioxide (5%). Cells were seeded on 96-well plates in the exponential growth phase using a cell suspension of 200 µL per well at a density between 250 and 1500 cells. After 24 h, the media were replaced with media of 200 µl containing serial concentrations of NPs (stocks prepared at 20 mg/mL in sterile phosphate-buffered saline). After 48 h, 72 h, or 7 days of treatment, the cells were subjected to a 3-(4,5-dimethylthiazol-2-yl)-2,5-diphenyltetrazolium bromide (MTT) assay, with 20 µL/well of MTT (Sigma-Aldrich, Burlington, MA, USA) solution at 5 mg/mL in phosphate-buffered saline. The cells were incubated for 3 h at 37 °C; after that, the MTT-containing media were removed, and the MTT reduced to purple formazan by living cells was solubilised by the addition of 200 µL/well of DMSO. After 1 h of incubation, the plates were measured at 560 nm using a scanning spectrophotometer microplate reader (Biochrom Asys UVM 340 Microplate Reader; ISOGEN, De Meern, The Netherlands). Quantities of the formazan product are directly related to the number of viable cells. At least three independent experiments, each performed in triplicate, were conducted in each case.

## 3. Results and Discussion

### 3.1. Synthesis and Functionalisation of Magnetic NPs (FeNPs)

Superparamagnetic NPs (Fe_3_O_4_ NPs) were synthesised using a co-precipitation method of FeCl_2_ and FeCl_3_ salts and their subsequent stabilisation by using different ligands with the aim of endowing Fe_3_O_4_ NPs with both colloidal stability and the ability to be encapsulated in the core of the micellar structures that drive the formation of MSNs. The optimal stabiliser ligands for the appropriate incorporation of Fe_3_O_4_ NPs into the h-MSNs were determined according to the morphological characteristics of the magnetic Fe_3_O_4_@MSNs observed from SEM images, as further discussed below. 

**Figure 1:**
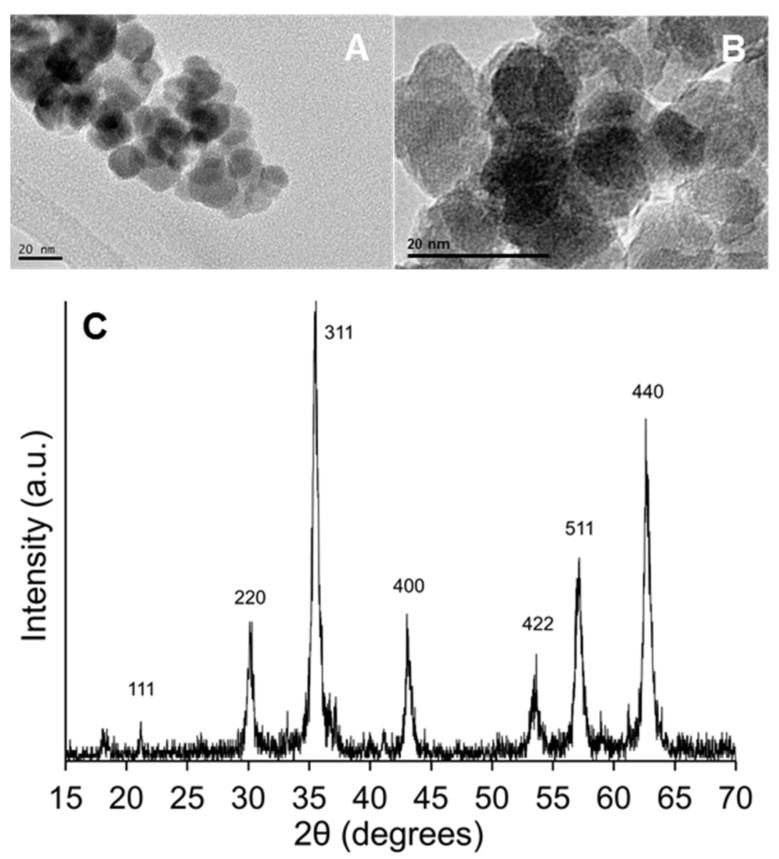
TEM images (**A**,**B**) and low-angle X-Ray diffraction pattern (**C**) of Fe_3_O_4_ NPs.

Figure 1A,B shows the SEM images of the synthesised Fe_3_O_4_ NPs at different magnifications. The sizes of the corresponding NPs were smaller than 20 nm. In addition, Figure 1C shows the low-angle XRD pattern of the Fe_3_O_4_ NPs, which allows the crystalline phase of magnetite to be identified, according to the assignation of the main reflections at 21.2, 30.1, 35.4, 43.0, 53.5, 57.0, and 62.6 (2θ degrees) for Miller indexes of 111, 220, 311, 400, 422, 511, and 440, respectively (JCPDS card No. 19-0629).

The synthesis of Fe_3_O_4_ NPs using the co-precipitation method leads to NPs that contain hydroxyl groups on their surface because of the basic media employed during the synthesis. The presence of hydroxyl moieties constitutes an advantage for the subsequent stabilisation by means of surface coating. Different stabiliser ligands were used, such as oleic acid, palmitic acid, butyric acid, citric acid, 3-aminopropyltrimethoxysilane (APS), and cetyltrimethylammonium bromide (CTAB); most of them are widely employed [34,35,36,37,38,39].

**Figure 2:**
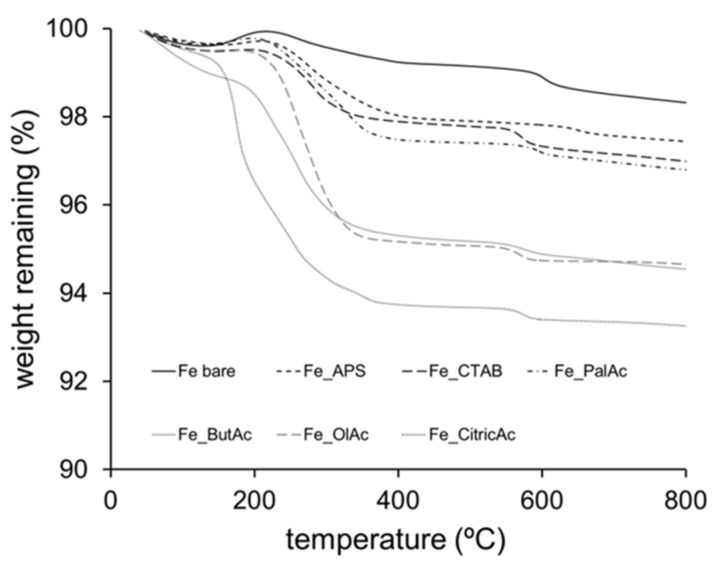
Thermal degradation of bare and functionalised Fe_3_O_4_ NPs with APS (Fe-APS), CTAB (Fe-CTAB), palmitic acid (Fe-PalAc), butyric acid (Fe-ButAc), oleic acid (Fe-OlAc), and citric acid (Fe-CitricAc).

Figure 2 shows the thermogravimetric analysis of Fe_3_O_4_ NPs. Bare as-made Fe_3_O_4_ NPs, without coating, showed a small degradation with a weight loss of 1% at high temperatures between 300 and 380 °C, corresponding to the dehydroxylation of the hydroxyl groups. In contrast, Fe_3_O_4_ NPs coated with stabiliser ligands exhibited higher weight losses, ranging from 2 to 7 wt.%. These results can be explained by the successful coating of Fe_3_O_4_ NPs. The interval of weight loss mediated by the efficacy of the functionalisation, corresponding to a maximum weight loss of 7 wt.%, can be attributed to the use of citric acid moieties. 

### 3.2. Synthesis of Magnetic MSNs (Fe_3_O_4_@MSNs)

The stability of Fe_3_O_4_ NPs can be improved by encapsulation with silica shells [55]. Thus, silica-coated Fe_3_O_4_ NPs were prepared by assembling the iron oxide NPs with stabiliser ligands followed by an oil-in-water silica sol-gel methodology, based on the drug-structure-directing agent (DSDA) concept previously reported [12]. The stabilised Fe_3_O_4_ NPs were combined with the DSDA and water in a molar ratio of 1:0.6:2000. Subsequently, after adding the co-structure-directing agent, oil-in-water emulsions were spontaneously formed, which spontaneously turned into spherical silica–surfactant aggregates after TEOS addition in a one-pot reaction [12]. The DSDA employed was N-decanoyl-L-tryptophan (NDLT), containing a hydrophobic chain of 10 carbon molecules and the model drug L-tryptophan.

**Figure 3:**
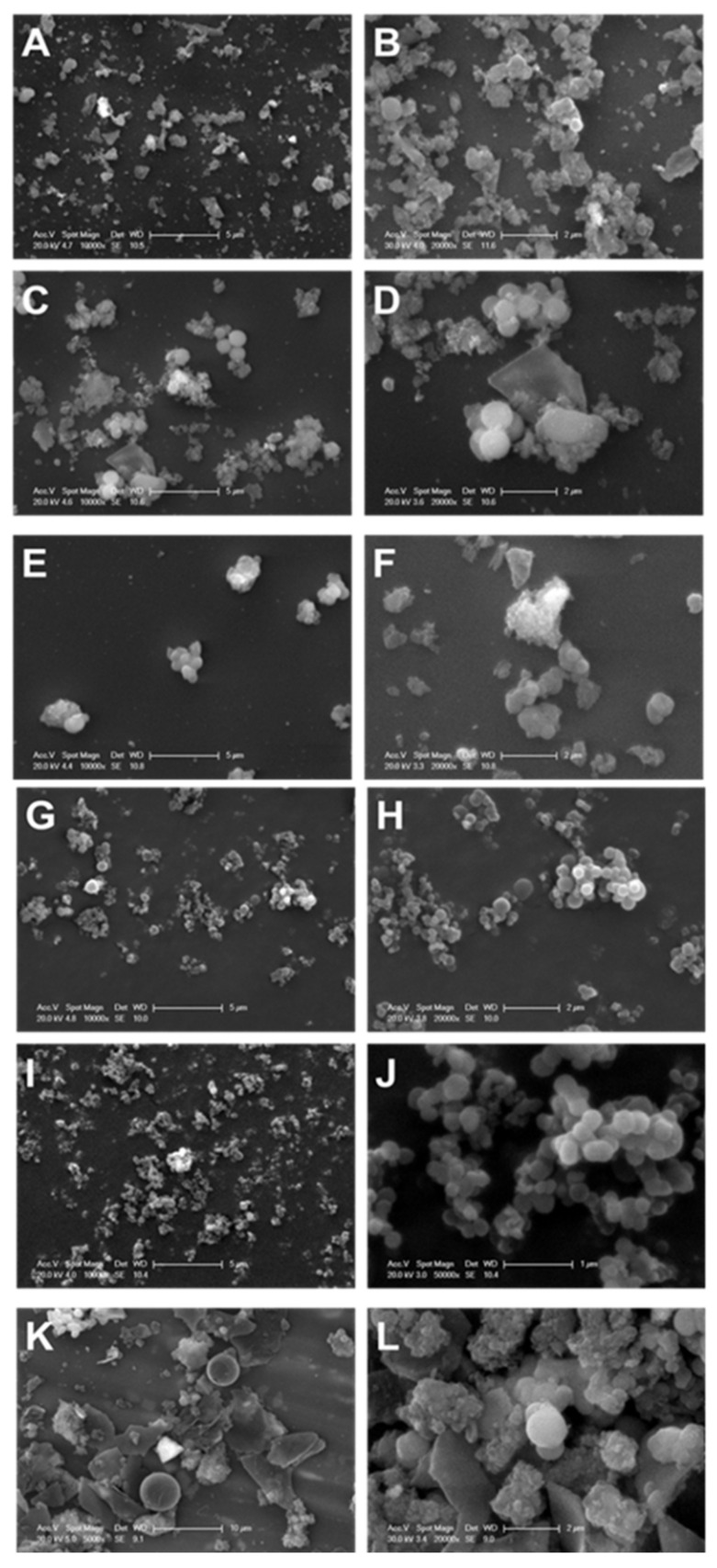
SEM images of the Fe_3_O_4_@MSN samples including FeNPs stabilised with oleic acid (**A**,**B**), palmitic acid (**C**,**D**), citric acid (**E**,**F**), butyric acid (**G**,**H**), CTAB (**I**,**J**), and APS (**K**,**L**).

The different morphological structures of the synthesised Fe_3_O_4_@MSNs, employing NDLT as a surfactant, were determined by SEM microscopy. Figure 3 shows that only the Fe_3_O_4_@MSNs prepared from Fe_3_O_4_ NPs stabilised with butyric acid and CTAB are characterised by a spherical shape without the presence of other undefined morphologies. In addition, the TEM images of these samples (results not shown) corroborated that only these two reagents are able to accomplish appropriate incorporation of Fe_3_O_4_ NPs into Fe_3_O_4_@MSNs (Figure 3). These results suggest that the correct incorporation of Fe_3_O_4_ NPs into Fe_3_O_4_@MSNs is accomplished by the hydrophobic nature of the functionalised Fe_3_O_4_ NPs, and the chain length of the reagent might have an influence as well.

**Figure 4:**
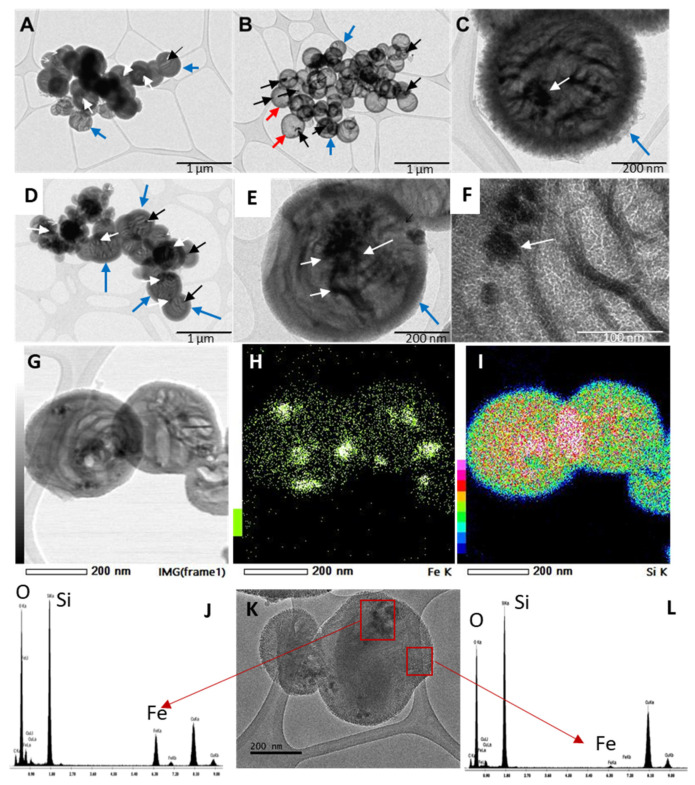
TEM images of the Fe_3_O_4_@MSN10-c (**A**–**C**) and Fe_3_O_4_@MSN10-b (**D**–**F**) samples. The black and white arrows correspond to magnetite nanoparticles. STEM image and elemental mapping of Fe and Si of the Fe_3_O_4_@MSN10-b (**G**–**I**). Elemental analysis in areas corresponding to small particles with dark contrast (**J**–**K**) and areas with no small particles and clear contrast (**K**–**L**).

Fe_3_O_4_@MSNs were synthesized by means of the combination of Fe_3_O_4_ NPs with the DSDA and water. The DSDA employed was NDLT (containing a hydrophobic chain of 10 carbon molecules). According to the above-mentioned study of the Fe_3_O_4_ NP stabilisers, the selected reagents for such functionalisation were CTAB and butyric acid, named as Fe_3_O_4_@MSN-b or Fe_3_O_4_@MSN-c, stabilised by butyric acid or CTAB, respectively. After the addition of Fe_3_O_4_ NPs and under mechanical stirring, a homogeneous brown solution was observed. Later, APS and TEOS were added, and clear brown precipitates were observed, suggesting good incorporation of the Fe_3_O_4_ NPs into the growing silica NPs. Some NPs with a dark black appearance, corresponding to Fe_3_O_4_ NPs that were not incorporated into the silica NPs, were observed in the TEM images. After washing, the excess Fe_3_O_4_ NPs were removed, and the resulting Fe_3_O_4_@MSNs were proven to have magnetic properties by the application of a magnet. Figure 4 shows the TEM images of the different Fe_3_O_4_@MSNs synthesised by employing both Fe_3_O_4_ NP stabiliser ligands. The Fe_3_O_4_@MSNs show the characteristic morphology of the NPs developed without the addition of Fe_3_O_4_ NPs [12]. Thus, the Fe_3_O_4_@MSNs exhibit a well-defined spherical shape (Figure 4A–F) with an average size of around 350 nm. The Fe_3_O_4_@MSNs contain inner cavities separated by internal mesoporous walls, and the NPs are surrounded by an external mesoporous shell. However, when CTAB was employed as a stabiliser ligand of Fe_3_O_4_ NPs, two different types of NPs were observed: hollow-shell NPs without mesoporous internal walls (Figure 4B, red arrows) and the characteristic NPs containing multiple cavities separated by mesoporous walls (Figure 4A–C, blue arrows). This phenomenon may be induced by the presence of some free CTAB and the ionic interactions that take place in the synthesis media because of the different species involved, which promote the formation of both types of NPs. When butyric acid was used as a functionalising agent, the NPs’ structures with several inner voids separated by a mesoporous wall and a thick mesoporous shell remained predominant (Figure 4D–F, blue arrows).

**Figure 5:**
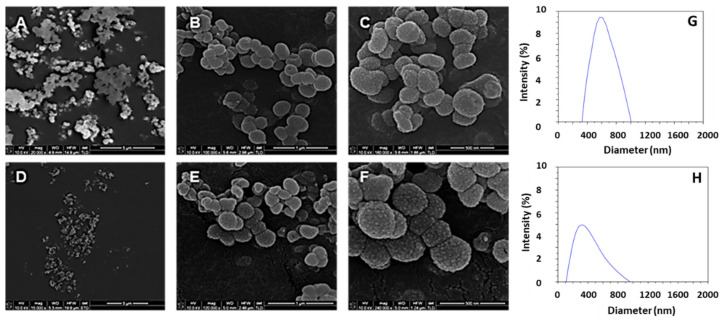
NanoSEM images of the surface of Fe_3_O_4_@MSNs samples: Fe_3_O_4_@MSN10-c (**A**–**C**), Fe_3_O_4_@MSN10-b (**D**–**F**). Particle size distributions: Fe_3_O_4_@MSN10-c (**G**), Fe_3_O_4_@MSN10-b (**H**).

The incorporation of Fe_3_O_4_ NPs into MSNs was successfully accomplished, regardless of the stabiliser ligand employed in the synthesis. Figure 4 shows the TEM images, and the presence of Fe_3_O_4_ NPs in the internal cavities of the NPs is clearly observed (black and white arrows in Figure 4A–F). To confirm the presence of Fe_3_O_4_ NPs into the internal cavities of the nanoparticles, an advanced STEM-XEDS analysis was performed. The elemental mapping based on energy dispersive X-rays (EDX) shows that the Fe atoms were successfully incorporated into the silica nanoparticles, which matches well with the small nanoparticles with dark contrast in the STEM images inside the silica cavities (Figure 4G–I). Moreover, TEM microanalysis in areas with small particles and dark contrast and areas with clear contrast was accomplished. The elemental analysis in areas corresponding to small particles with dark contrast shows the characteristic peaks of Fe, corroborating the presence of Fe_3_O_4_ NPs (Figure 4J), while in the clear areas, no evidence or negligible presence of Fe was confirmed (Figure 4L). Additionally, the different Fe_3_O_4_@MSNs synthesised showed a similar external morphology. The SEM images of Figure 5 show the nearly spherical geometry of the NPs, with a remarkable rough surface and a raspberry-like morphology with protrusions or depressions. This feature is very interesting considering the special adhesion and superior cell uptake properties of NPs with rough surfaces, compared with NPs with smooth surfaces, as a result of the lower repulsive interactions with cell membranes [56,57,58,59,60].

**Figure 6:**
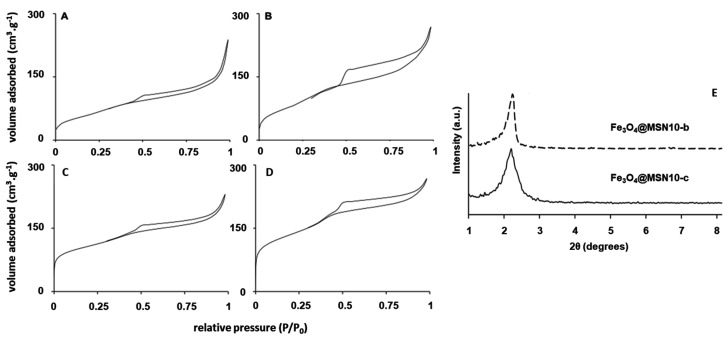
Nitrogen adsorption–desorption isotherms of extracted (**A**,**B**) and calcined (**C**,**D**) Fe_3_O_4_@MSNs samples: Fe_3_O_4_@MSN10-c (**A**,**C**), Fe_3_O_4_@MSN10-b (**B**,**D**). Low-angle X-ray diffraction patterns of the Fe_3_O_4_@MSNs samples (**E**).

Figure 6 shows the low-angle XRD diffractograms. The patterns show the typical characteristic diffraction peaks corresponding to mesostructured silica materials, while the wide-angle XRD patterns (not shown) show no evidence of a magnetite phase, probably because the iron oxide NPs were within the MSNs and the diffraction peaks are not discerned. The N_2_ adsorption–desorption isotherms (Figure 6) of the Fe_3_O_4_@MSNs after the extraction and calcination processes show typical type IV curves with capillary condensation steps at a relative pressure of 0.35−0.4, corroborating the presence of mesopores. The Fe_3_O_4_@MSNs exhibit a type H_3_ hysteresis loop, which might be attributed to the effect of the internal cavities or hollows [12,61,62]. The pore size distribution was calculated by means of the non-local density functional theory (NLDFT) model, obtaining a wide pore distribution as a result of the contribution of the different pore and intra-void sizes of the NPs (Table 1), with a main contribution around 3.0 nm. 

Table 1 summarises the BET surface area and the total pore volume. The S_BET_ of the Fe_3_O_4_@MSNs was higher when butyric acid instead of CTAB was employed as a functionalising agent of the Fe_3_O_4_ NPs (394 m^2^/g vs. 227 m^2^/g). This fact may be associated with the higher stability of Fe_3_O_4_ NPs functionalised using butyric acid, probably due to the smaller size of the hydrocarbon chain with respect to CTAB. The calcination process led to a remarkable increment in S_BET_ for all the samples. This can be explained by shell shrinkage as well as by a reduction in internal mesoporous walls upon calcination. 

**Figure 7:**
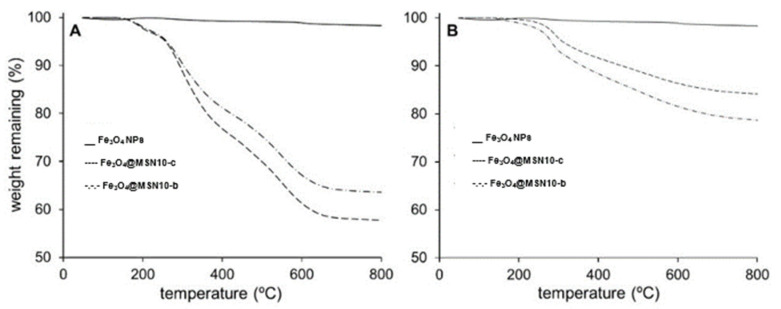
Thermal degradation of bare Fe_3_O_4_ NPs and Fe_3_O_4_@MSNs samples, as made (**A**) and after the extraction of DSDA and FeNPs (**B**).

The thermal degradation of the samples (Figure 7) provided information about the organic content (amounts of DSDA and APS). The thermograms are characterised by several overlapping degradation stages between 200 and 700 °C, associated with the removal of the APS and DSDA chains. The residual content in the as-made NPs (Figure 7A) was similar in the different NPs (between 55 and 65% of their initial weight, which corresponds to the percentage in weight of the silica framework and Fe_3_O_4_ NPs). On the other hand, the residual content of the extracted materials (Figure 7B) was between 80 and 90%, which corresponds to the weight (%) of the silica framework after the extraction.

### 3.3. DSDA Delivery and Cell Viability

**Figure 8:**
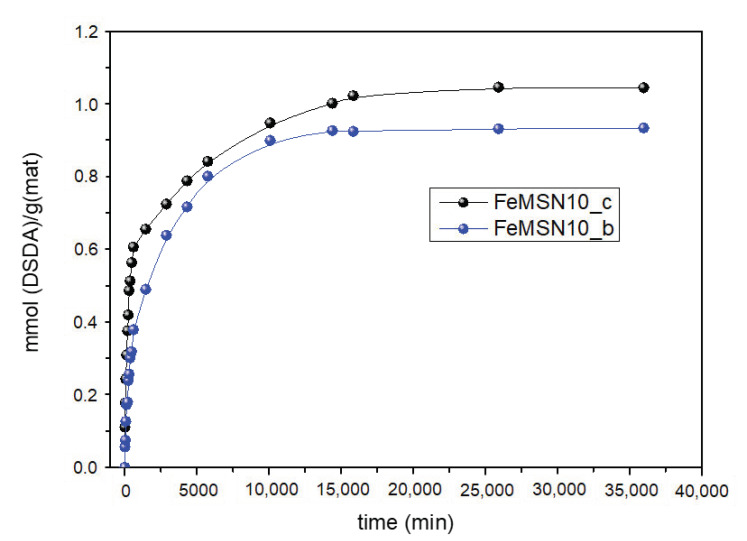
DSDA release as a function of time from the Fe_3_O_4_@MSNs synthesised.

The delivery of DSDA from the Fe_3_O_4_@MSNs was conducted in a solution of 0.1 M NaHCO_3_ with pH 7.4. Figure 8 shows a sustained release of DSDA during a remarkable period of 20 days. The trend is characterised by a linear release at short times and a stabilisation of the DSDA release at longer times. On the other hand, the release does not depend on the functionalisation of Fe_3_O_4_ NPs. 

**Figure 9:**
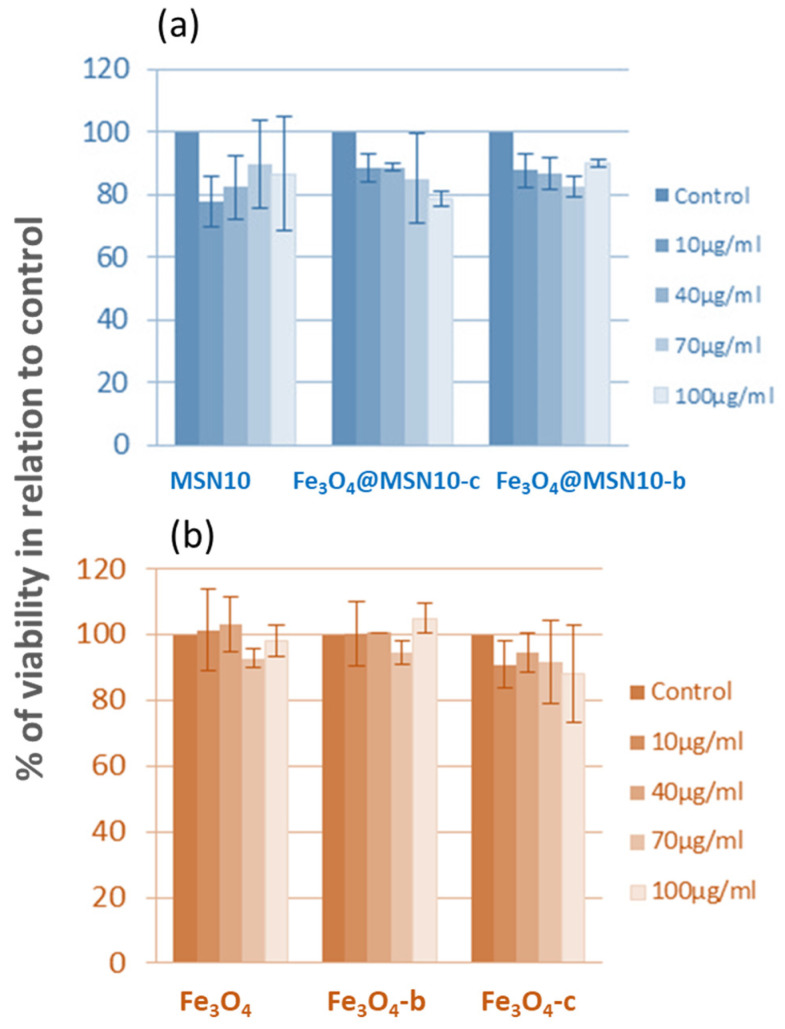
Influence of Fe_3_O_4_@MSNs (**a**) or Fe_3_O_4_ NPs (**b**) on cell viability in DLD1 human colon cancer cells. Cells were treated with increasing concentrations of six different nanoparticles over 48 h; cell viability was determined by MTT assay. Bars represent the mean values obtained from at least three independent experiments, each performed in triplicate.

The possible cytotoxic effects of the functionalised Fe_3_O_4_ NPs and Fe_3_O_4_@MSNs employing NDLT as a surfactant were addressed using DLD1 human colon cancer cells. The viability of these cells was evaluated by carrying out MTT assays after 48 h of incubation and at different concentrations of NPs in the culture medium (Figure 9). Figure 9 indicates that the different NP systems showed no cytotoxic effect. Qualitative similar results were also observed in an additional human colon cancer cell line, HCT-116.

**Figure 10:**
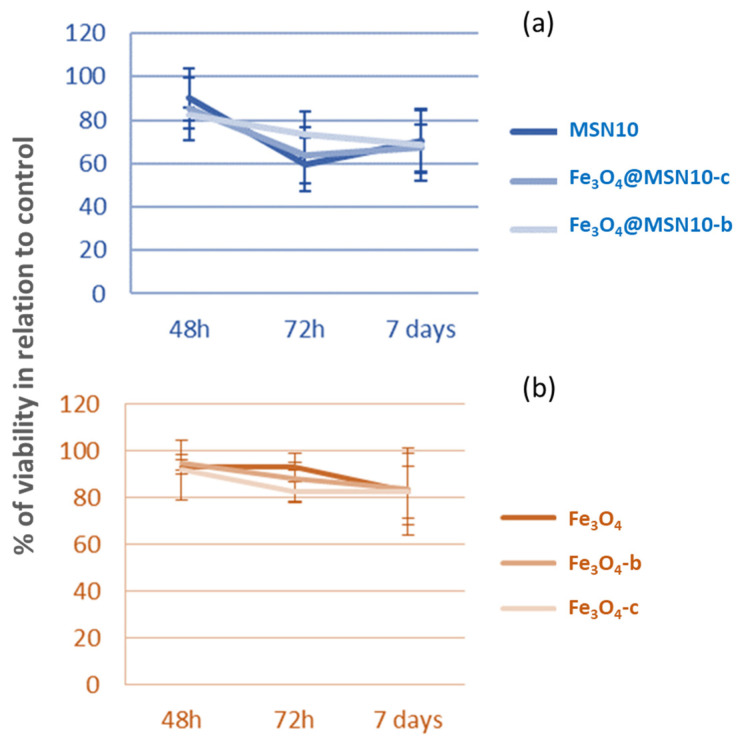
Influence of 70 µg/mL of Fe_3_O_4_@MSNs (**a**) or Fe_3_O_4_ NPs (**b**) on cell viability in DLD1 colon cancer cells at different times. Cells were treated with 70 µg/mL of the six nanoparticles and viability was measured by MTT assay at 48 h, 72 h, or 7 days.

The cytotoxic effect was studied even at a concentration of NPs in the culture medium of 70 µg/mL and after 7 days of incubation (Figure 10). Even after 7 days, none of the NPs showed cytotoxicity, and only a slight effect on cell growth was observed, which was not dependent on time or dose. The Fe_3_O_4_ NPs had a low antiproliferative activity against DLD1 colon cancer cells, independently of employing bare Fe_3_O_4_ NPs or functionalised Fe_3_O_4_ NPs.

## 4. Conclusions

In summary, hollow-shell MSNs with a raspberry-like rough surface and iron oxide magnetite inside have been successfully synthesised through an oil-in-water emulsion method based on the DSDA concept. The DSDA is composed of the model drug L-tryptophan modified with decanoic aliphatic hydrocarbon chains, which are responsible for the structure of the h-MSNs but also impart intrinsic pharmacological activity to the h-MSNs. Iron oxide NPs whose external surfaces were modified by the ligand stabilisers CTAB and butyric acid were also added to the synthesis media to add magnetic properties. The one-pot synthesis led to all-in-one NPs with a well-defined hollow-shell structure with a rough morphology and with Fe_3_O_4_ NPs incorporated into the inner cavities of the h-MSNs, separated by internal mesoporous walls and an outer shell with mesopores of 3 nm containing the model drug tryptophan. The obtained Fe_3_O_4_@MSNs possess large surface areas of up to 500 m^2^·g^−1^ and a pore volume of 0.4 cm^3^·g^−1^. Additionally, the in vitro delivery studies showed a sustained release of DSDA tryptophan from Fe_3_O_4_@MSNs for 20 days. Cell viability experiments indicated that Fe_3_O_4_@MSNs showed no cytotoxicity and only low antiproliferative activity against colon cancer cells. Because of the magnetic properties, rough surface, and open and accessible mesopore channels that can be loaded with different drugs, it is expected that these Fe_3_O_4_@MSNs may find applications in drug delivery in cancer therapy.

## Figures and Tables

**Table 1:** Textural properties of the extracted and calcined Fe_3_O_4_@MSNs.

DSDA Removal	Sample	Vp (cm^3^/g)	S_BET_ (m^2^/g)	PSD (nm) *	Fe_ICP_ (wt%)
**Extracted in EtOH/HCl**	Fe_3_O_4_@MSN10-c	0.232	227.17	2.1–47.1 (2.9)	
	Fe_3_O_4_@MSN10-b	0.266	394.32	2.0–43.1 (2.9)	
**Calcined**	Fe_3_O_4_@MSN10-c	0.389	392.3	1.7–4.0 (3.0)	2.5
	Fe_3_O_4_@MSN10-b	0.335	507.96	1.2–3.9 (3.0)	2.7

* Range of the pore diameter and the most frequent pore width (between brackets).
